# Post‐Neoadjuvant Chemotherapy Axillary Ultrasound in cN+ Breast Cancer: Can It Reliably Support de‐Escalation of Axillary Surgery?

**DOI:** 10.1002/cam4.71759

**Published:** 2026-03-27

**Authors:** Sara Albasini, Camilla Rossetti, Roberta Brancaccio, Elena Orvieto, Daniela Bossi, Matilde Pelizzola, Marta Truffi, Serena Mazzucchelli, Valentina Zanella, Arianna Bonizzi, Carlo Morasso, Fabio Corsi

**Affiliations:** ^1^ Breast Unit Istituti Clinici Scientifici Maugeri IRCCS Pavia Italy; ^2^ General Surgery Residency Program University of Milan Milan Italy; ^3^ Nanomedicine and Molecular Imaging LabIstituti Clinici Scientifici Maugeri IRCCS Pavia Italy; ^4^ Department of Biomedical and Clinical Sciences University of Milan Milan Italy

**Keywords:** axillary dissection, axillary ultrasound, breast cancer, lymph node staging, neoadjuvant chemotherapy, sentinel lymph node biopsy, surgical de‐escalation

## Abstract

**Background:**

Neoadjuvant chemotherapy (NAC) in up to 40%–60% of node‐positive (cN+) breast cancer patients allows nodal pathological complete response, particularly in Her2+ and triple‐negative subtypes. Accurate post‐NAC axillary restaging is therefore critical to identify candidates for surgical de‐escalation. Axillary ultrasound (AUS) remains the most widely used tool, but prior studies report highly variable performance, with false‐negative rates approaching 25%, raising concerns about its reliability as a standalone guide.

**Methods:**

We retrospectively evaluated 413 patients with biopsy‐proven cN+ breast cancer treated with NAC (2000–2024). All underwent AUS before surgery. AUS results (ycN0 vs. ycN+) were compared with final pathology (ypN0 vs. ypN+). Diagnostic performance was assessed, and predictors of false‐negative AUS were identified using logistic regression.

**Results:**

AUS classified 51.6% of patients as ycN0, whereas only 45% achieved nodal pathologic complete response. Overall, AUS demonstrated 68% sensitivity, 76% specificity, 72% accuracy, positive predictive value 78%, and negative predictive value 66%. Performance differed by subtype: accuracy was highest in ER‐/Her2 tumors (75%), but markedly lower in Her2+ (69%) and lobular carcinoma (67%). On multivariate analysis, absence of breast clinical complete response (OR 2.38, 95% CI [1.14–4.93]) independently predicted false‐negative AUS findings.

**Conclusions:**

AUS following NAC provides only moderate accuracy, with a clinically relevant risk in patients lacking breast response. AUS alone should not determine omission of axillary surgery. A multimodal, biology‐informed strategy combining AUS with other imaging techniques is needed to safely guide axillary de‐escalation and minimize both overtreatment and undertreatment.

## 1 Introduction

Neoadjuvant chemotherapy (NAC) is a cornerstone in the treatment of locally advanced and biologically aggressive breast cancer, offering a dual benefit: a real‐time assessment of therapeutic response and tumor downstaging [1, 2, 3]. One of its most clinically relevant effects is the potential to convert axillary lymph node involvement from positive to negative, allowing for less extensive surgery and reduced morbidity [2, 4]. For patients initially presenting with node‐positive disease (cN+), accurate post‐NAC axillary restaging is essential to guide de‐escalated approaches, including sentinel lymph node biopsy (SLNB) or targeted axillary dissection (TAD) [4, 5]. Multiple previous studies showed how non‐invasive axillary re‐staging imaging after NACT is challenging and it constitutes a significant unmet clinical need in breast cancer management [6, 7].

Axillary ultrasound (AUS) remains the most widely used imaging modality for post‐NAC nodal assessment due to its accessibility and low cost [8, 9]. However, its diagnostic performance is not very effective, especially in the context of chemotherapy‐induced tissue changes that can obscure residual disease or mimic benign nodal features [10, 11]. Reported positive predictive values (PPV) vary widely, and studies suggest that factors such as tumor subtype, grading, pathological complete response (pCR), and operator experience may substantially influence AUS accuracy [12, 13]. Recent studies found that AUS missed nodal metastases in many cases deemed ycN+ [14, 15], raising concerns about its reliability as a standalone decision‐making tool.

Retrospective series and the prospective AXSANA registry have reported high PPV but modest sensitivity and negative predictive value (NPV) for AUS after NAC, with particularly poor NPV in luminal tumors and better performance in HER2‐positive and triple‐negative disease, which are more likely to achieve axillary pCR. Subtype‐stratified analyses by Di Micco [16] have shown that a positive AUS is especially informative in luminal cancers, whereas a negative AUS is more reliable in HER2‐enriched and triple‐negative tumors.

Other imaging techniques such as magnetic resonance imaging (MRI) and positron emission tomography‐computerized tomography (PET‐CT) offer improved soft tissue resolution and functional assessment, respectively, and have demonstrated similar diagnostic accuracy in selected post‐NAC settings [17, 18, 19]. Besides, in recent years, novel axillary surgical strategies have been developed to overcome the limitations of both AUS and conventional SLNB. Among them, TAD has gained prominence as a method to improve staging accuracy after NAC [20, 21]. The prospective AXSANA study, one of the largest international multicenter efforts in this field, has provided critical insights into the practical challenges of axillary surgery after NAC [22]. Importantly, AXSANA highlighted the issue of lost markers after NAC, which can compromise the accuracy of targeted retrieval and complicate nodal staging [23]. These findings emphasize that technical factors, in addition to imaging accuracy, may influence surgical outcomes and must be considered when evaluating the reliability of AUS and other staging methods.

Despite AUS being entrenched in clinical pathways, its actual predictive value for residual nodal disease post‐NAC in biopsy‐confirmed cN+ patients remains poorly defined [11].

This study aims to assess the PPV of AUS following NAC in a large, single‐center cohort of patients with initially node‐positive breast cancer. By quantifying its diagnostic accuracy, we seek to guide more precise axillary surgical strategies and challenge assumptions surrounding the reliability of AUS in this critical clinical context.

## 2 Materials and Methods

### 2.1 Study Population

This is a retrospective, single‐center study conducted at the Breast Unit of Istituti Clinici Scientifici Maugeri IRCCS in Pavia, Italy. Data were collected in accordance with a study protocol approved by the Institutional Ethics Committee (No. 2590/2021). All relevant clinical and histopathological information, including data on neoadjuvant treatment, diagnostic assessments, and surgical procedures, were collected. We included patients diagnosed with invasive breast cancer cN+ axillary disease confirmed by AUS with or without biopsy/cytology/PET‐CT/MRI, who received NAC between January 2000 and March 2024.

Inclusion criteria were: confirmed invasive breast carcinoma, cN+ disease prior to NAC, full administration of NAC, post‐NAC AUS performed before surgery.

Preoperative and postoperative AUS evaluation was performed by a Breast Unit radiology team specialized in breast imaging with years of experience.

Given the extended timeframe of the study, axillary management evolved over time, shifting from routine axillary dissection (ALND) in the initial phases to SLNB alone. In all cases of sentinel lymph node positivity, patients subsequently underwent axillary lymph node dissection. Patients with inflammatory breast cancer, distant metastases at diagnosis, incomplete NAC or missing radiologic or pathologic data were excluded from analysis.

### 2.2 Evaluation of Clinical and Pathological Response

AUS examinations were conducted by experienced breast radiologists using high‐frequency linear transducers. Post‐NAC AUS findings were categorized as ycN+ if any morphologically suspicious lymph nodes were identified, including focal or diffuse cortical thickening > 3 mm, loss of fatty hilum, or rounded shape. Patients without these features were classified as ycN0. These criteria remained consistent throughout the entire study period, ensuring uniform evaluation parameters for all patients included in the study.

Since NAC regimens differ substantially across biomolecular subtype and influence both pCR and nodal clearance, treatment regimens were included as an adjustment variable in the multivariable logistic regression model. Regimens were categorized according to standard subtype‐specific therapeutic protocols. This approach allowed us to account for the potential confounding effect of treatment intensity and subtype‐specific chemosensitivity (Table 3).

Following NAC and imaging reassessment, all patients underwent surgical treatment, including breast surgery, SLNB and/or ALND. Decision regarding ALND or SLNB were made according to international guidelines and multidisciplinary team assessment. Final axillary nodal status (ypN+ or ypN0) was determined through standard histopathologic evaluation. Axillary pCR was defined as no micro‐ or macrometastases in any excised lymph node (ypN0/ITC+).

### 2.3 Endpoints and Study Design

The primary endpoint was the evaluation of the PPV and NPV of post‐NAC AUS. PPV was defined as the proportion of patients classified as ycN+ who were found to have residual nodal metastases (ypN+) on final pathology, while NPV was defined as the proportion of patients classified as ycN0 who had no residual nodal metastases (ypN0). Secondarily, we evaluated whether relevant clinical or histopathological factors could predict axillary nodal positivity following a ycN0 assessment on post‐NAC AUS (false negative axillary status by AUS). Clinical, radiologic, and pathological data were retrospectively collected from institutional electronic medical records.

### 2.4 Statistical Analysis

Variables were reported as medians, interquartile range, and ranges, or as absolute numbers and percentages. Categorical variables were compared using the χ2 test or Fisher exact tests as appropriate, while continuous variables were compared using the non‐parametric Wilcoxon test. To evaluate the impact of clinical and histopathological variables on axillary status prediction by AUS, a logistic regression analysis was performed. Variables showing significant associations in the univariate analysis, expressed as odds ratios (OR) with 95% confidence intervals (CI), were subsequently included in a multivariate model to identify potential independent effects. To better evaluate diagnostic performance, the Receiver Operating Characteristic (ROC) curve and the Area Under the Curve (AUC) were reported for the models for which this was applicable. Statistical significance was set at *p* < 0.05 (two‐tailed). Data analysis was performed using SAS software (v. 9.4, SAS Institute Inc., Cary, USA).

## 3 Results

### 3.1 Baseline Characteristics

A total of 413 patients with invasive breast cancer and biopsy‐proven node‐positive (cN+) axillary involvement at diagnosis were included in the analysis. All patients underwent NAC followed by AUS before surgery, with missing data accounting for less than 4%. As reported in Table 1, the median age at diagnosis was 53 years (IQR 19 [25–83]), and the median tumor size on baseline imaging was 30 mm (IQR 15 [5–130]). Most patients presented with cT2 tumors (56.9%) and cN1 nodal involvement (77.5%). Invasive ductal carcinoma was the most frequent histologic subtype (86.7%), and the predominant biomolecular subtype was ER+/Her2‐ (44.8%), followed by Her2+ (40.9%) and a smaller proportion of triple‐negative cases (14.3%). Following NAC, axillary ultrasound reassessment classified 51.6% of patients (*n* = 213) as ycN0 and 48.4% (*n* = 200) as ycN+. However, the final pathological evaluation revealed that 45% (*n* = 186) of the entire cohort achieved ypN0, while 55% (*n* = 227) still harbored status residual nodal disease.

**TABLE 1:** Baseline characteristics of the cohort and correlation with final histopathological findings.

	cN+ patients before NAC (*n* = 413)	ypN0 (*n* = 186)	ypN+ (*n* = 227)	*p*
Age at diagnosis (years)*	53 (19) [25–83]	53 (18) [25–83]	53 (19) [27–81]	0.46
Patients' Body Mass Index (BMI)*	24 (7) [13–46]	24 (7) [13–46]	25 (6) [15–39]	0.15
Breast lesion size on imaging (mm)*	30 (15) [5–130]	30 (18) [5–130]	35 (23) [7–120]	0.003
Multifocal disease on imaging				
No	290 (70.4)	132 (71.4)	158 (69.6)	0.70
Yes	122 (29.6)	53 (28.7)	69 (30.4)	
Pre‐treatment clinical T stage				
cT1	75 (18.3)	43 (23.5)	32 (14.2)	0.01
cT2	232 (56.9)	105 (57.4)	127 (56.4)	
cT3	37 (9.1)	16 (8.7)	21 (9.3)	
cT4	64 (15.7)	19 (10.4)	45 (20)	
Pre‐treatment clinical N stage				
cN1	320 (77.5)	157 (84.4)	163 (71.8)	0.005
cN2	74 (17.9)	21 (11.3)	53 (23.4)	
cN3	19 (4.6)	8 (4.3)	11 (4.8)	
Histological type (CB)				
Ductal	358 (86.7)	173 (93)	185 (81.5)	0.0006
Lobular	55 (13.3)	13 (7)	42 (18.5)	
Grading (CB)				
G1	3 (0.7)	1 (0.5)	2 (0.9)	0.002
G2	250 (62.5)	95 (53.4)	155 (69.8)	
G3	147 (36.8)	82 (46.1)	65 (29.3)	
Biomolecular subtype (CB)				
Her2+	169 (40.9)	103 (55.4)	66 (29.1)	< 0.0001
ER+/Her2—	185 (44.8)	50 (26.9)	135 (59.5)	
ER‐/Her2—	59 (14.3)	33 (17.7)	26 (11.4)	
Ki67 (CB)				
< = 14%	109 (26.4)	36 (19.3)	73 (32.2)	0.003
> 14%	304 (73.6)	150 (80.7)	154 (67.8)	
NAC				
Anthracyclines/FEC (Type1)	113 (27.6)	27 (14.7)	86 (38.1)	< 0.0001
Anthracyclines/FEC + Taxanes (Type2)	146 (35.6)	59 (32.1)	87 (38.5)	
Anthr/FEC + Taxanes+ anti‐Her2 (Type3)	124 (30.2)	84 (45.7)	40 (17.7)	
Others (Type 4)	27 (6.6)	14 (7.6)	13 (5.8)	
Post NAC breast cCR				
No	302 (79.7)	106 (63.9)	196 (92)	< 0.0001
Yes	77 (20.3)	60 (36.1)	17 (8)	
Clinical evaluation after NAC				
Negative	242 (63.7)	149 (84.7)	93 (45.6)	< 0.0001
Positive	138 (36.3)	27 (15.3)	111 (54.4)	
AUS after NAC				
Negative	213 (51.6)	141 (75.8)	72 (31.7)	< 0.0001
Positive	200 (48.4)	45 (24.2)	155 (68.3)	
Type of breast surgery				
Breast‐conserving surgery	196 (47.5)	99 (53.2)	97 (42.7)	0.03
Total mastectomy	217 (52.5)	87 (46.8)	130 (57.3)	
Breast pCR				
No	263 (63.7)	70 (37.6)	193 (85)	< 0.0001
Yes	150 (36.3)	116 (62.4)	34 (15)	
Axillary status				
ypN0	186 (45)	—	—	
ypN+	227 (55)	—	—	

*Note:* Values are *n* (%) unless otherwise indicated; values.

Abbreviations: AUS, Axillary ultrasound; CB, Core biopsy; cCR, Clinical complete response; ER, Estrogen; NAC, Neoadjuvant chemotherapy; pCR, Pathological complete response.

* Are median (i.q.r.) [range].

Upon stratifying the cohort into ypN0 and ypN+ groups, Her2+ patients displayed the highest probability of achieving ycN0 axillary status after NAC (62.1%), consistent with the known chemosensitivity of the subgroup. By contrast, only 43.2% of ER+/Her2 and 47.5% of triple‐negative patients achieved ycN0 status (*p* = 0.0001).

On the entire cohort, NAC regimens were distributed as follows: 27.6% received anthracycline‐based therapy (Type 1), 35.6% anthracycline with Taxanes (Type 2), 30.2% Taxanes‐anthracycline regimens combined with anti‐Her2 therapy (Type 3), and 6.6% other regimens (Type 4). Nodal response differed markedly across these groups, with the highest ypN0 rates observed in Type 3 regimens (45.7%), compared with 32.1% in Type 2, 14.7% in Type 1, and 7.6% in Type 4. These findings reflect the expected subtype‐driven differences in pCR and highlight treatment regimen as an important factor influencing nodal clearance.

### 3.2 Diagnostic Performance of AUS

Diagnostic performance of post‐NAC ultrasound evaluation was reported in Table 2. Overall, AUS demonstrated sensitivity of 68%, specificity of 76%, accuracy of 72%, PPV of 78% and NPV of 66%.

**TABLE 2:** Diagnostic performance of post‐NAC ultrasound evaluation overall and across different biological subtypes of breast cancer.

	AUS after NAC		Biomolecular subtype (CB)						Histological type (CB)			
			Her2+		ER+/Her2‐		ER‐/Her2‐		Ductal		Lobular	
	ycN0	ycN+	ycN0	ycN+	ycN0	ycN+	ycN0	ycN+	ycN0	ycN+	ycN0	ycN+
ypN0	141	45	78	25	40	10	23	10	131	42	10	3
ypN+	72	155	27	39	40	95	5	21	57	128	15	27
	Sensitivity: 68%		Sensitivity: 59%		Sensitivity: 70%		Sensitivity: 81%		Sensitivity: 69%		Sensitivity: 64%	
	Specificity: 76%		Specificity: 76%		Specificity: 80%		Specificity: 70%		Specificity: 76%		Specificity: 77%	
	Accuracy: 72%		Accuracy: 69%		Accuracy: 73%		Accuracy: 75%		Accuracy: 72%		Accuracy: 67%	
	PPV: 78%		PPV: 61%		PPV: 90%		PPV: 68%		PPV: 75%		PPV: 90%	
	NPV: 66%		NPV: 74%		NPV: 50%		NPV: 82%		NPV: 70%		NPV: 40%	

*Note:* Values are *n* (%) unless otherwise indicated.

Abbreviations: AUS, Axillary ultrasound; CB, Core biopsy; ER, Estrogen; NAC, Neoadjuvant chemotherapy.

**FIGURE 1:**
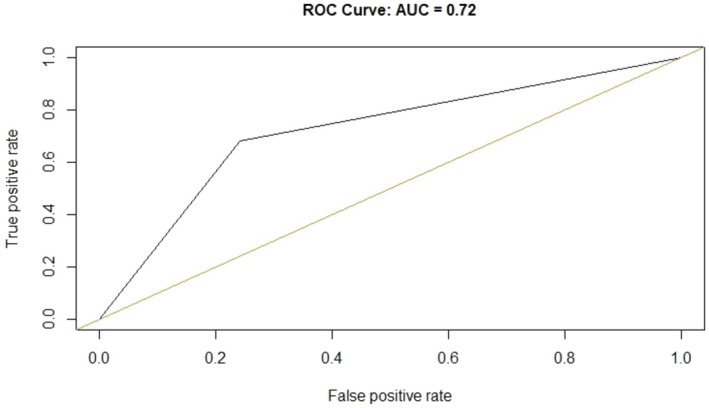
ROC (Receiver operating characteristic) curve and relative area under the curve (AUC) for the diagnostic performance of post‐NAC ultrasound evaluation of axilla.

For the post‐NAC AUS, the corresponding ROC curve is reported in Figure 1. The AUC was found to be 0.72 too, with a 95% CI [0.67–0.77].

Stratifying biomolecular subtype, Er‐/Her2 tumors showed the best diagnostic accuracy (75%). Her2+ tumors fared worse, with an accuracy of only 69%, despite their higher rate of nodal eradication under NAC.

From a histological standpoint, ductal carcinomas achieved a higher NPV (70%), while lobular carcinomas showed a strikingly low NPV (40%).

### 3.3 Clinical and Histopathological Predictors of False‐Negative Axillary Status After ycN0 Assessment by Post‐NAC AUS

In the univariate analysis reported in Table 3, pre‐treatment clinical T stage did not show a significant association with the probability of false negative lymph nodes (OR = 0.73, 95% CI [0.36–1.48], *p* = 0.38) as well as tumor grading (OR = 1.21, 95% CI [0.67–2.19], *p* = 0.53) and Ki67 proliferation index (OR = 1.48, 95% CI [0.77–2.87], *p* = 0.24).

**TABLE 3:** Univariate and multivariate analyses to identify predictors of false negative lymph nodes (only ycN0 patients at AUS).

	False negative vs. true negative lymph nodes (computed the probability of false negative lymph nodes)					
	Univariate			Multivariate		
	OR	95% CI	*p*	OR	95% CI	*p*
Pre‐treatment clinical T stage						
cT1/cT2	0.73	0.36–1.48	0.38	—	—	—
cT3/cT4	Ref.	—	—	—	—	—
Histological type (CB)						
Ductal	0.29	0.12–0.68	0.005	0.61	0.23–1.60	0.31
Lobular	Ref.	—	—	Ref.	—	—
Grading (CB)						
G1/G2	1.21	0.67–2.19	0.53	—	—	—
G3	Ref.	—	—	—	—	—
Biomolecular subtype (CB)						
ER+/Her2—	2.89	1.55–5.37	0.0008	1.45	0.43–4.97	0.55
ER‐/Her2—	1.59	0.55–4.60	0.39	0.46	0.11–1.92	0.29
Her2+	Ref.	—	—	Ref.	—	—
NAC						
Anthracyclines/FEC (Type1)	2.43	1.08–5.49	0.03	1.45	0.41–5.11	0.56
Anthracyclines/FEC + Taxanes (Type2)	2.22	1.13–4.38	0.02	2.03	0.55–7.39	0.28
Others (Type4)	1.59	0.44–5.83	0.48	0.93	0.17–5.06	0.94
Anthr/FEC + Taxanes+ anti‐Her2 (Type3)	Ref.	—	—	Ref.	—	—
Ki67 (CB)						
< = 14%	1.48	0.77–2.87	0.24	—	—	—
> 14%	Ref.	—	—	—	—	—
Post NAC breast cCR						
No	2.86	1.44–5.70	0.003	2.38	1.14–4.93	0.02
Yes	Ref.	—	—	Ref.	—	—

Abbreviations: CB, Core biopsy; cCR, Clinical complete response; ER, Estrogen; NAC, Neoadjuvant chemotherapy.

Histological type was significantly associated with axillary status: ductal carcinomas had a lower likelihood of nodal positivity compared with lobular tumors (OR = 0.29, 95% CI [0.12–0.68], *p* = 0.005).

Biomolecular subtypes showed a strong influence on nodal positivity. Compared to Her2+ tumors, ER+/Her2‐ tumors were associated with a higher risk of axillary involvement (OR = 2.89, 95% CI [1.55–5.37], *p* = 0.0008), whereas ER‐/Her2 tumors did not show a significant association (OR = 1.59, 95% CI [0.55–4.60], *p* = 0.39). A similar pattern was observed across neoadjuvant regimens: compared with patients who received Type 3 treatment, those who received Type 1 (OR = 2.43, 95% CI [1.08–5.49], *p* = 0.03) or Type 2 (OR = 2.22, 95% CI [1.13–4.38], *p* = 0.02) had a higher risk of false‐negative lymph nodes, whereas no significant difference was observed with Type 4 (*p* = 0.48). Post‐NAC breast clinical complete response (cCR) was significantly associated with axillary status. Patients who did not achieve cCR had a higher likelihood of residual nodal disease (OR = 2.86, 95% CI [1.44–5.70], *p* = 0.003) compared with those who achieved cCR.

The multivariate analysis, also presented in Table 3, identified one independent predictor of false‐negative AUS findings among ycN0 patients, selected from the three variables that were statistically significant in the univariate analysis. Lack of complete clinical response in the breast (cCR) strongly predicted residual nodal disease despite negative AUS (OR = 2.38, 95% CI [1.14–4.93], *p* = 0.02). Lobular histology, biomolecular subtypes, and NAC regimens lost significance in adjusted models.

**FIGURE 2:**
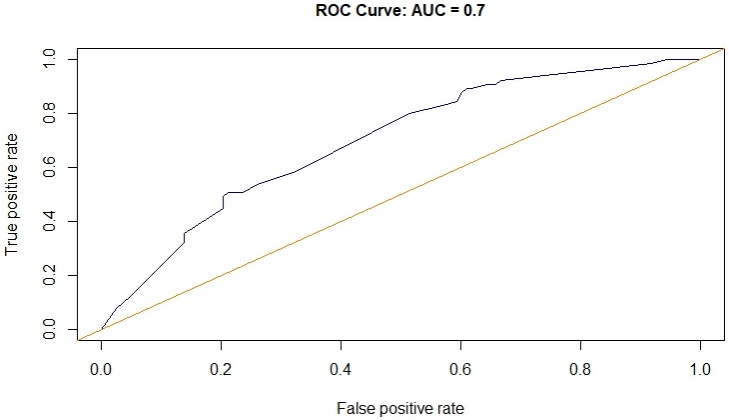
ROC (Receiver operating characteristic) curve and relative area under the curve (AUC) for multivariate model.

For the selected model, the corresponding ROC curve is reported in Figure 2. The AUC was found to be 0.70, with a 95% CI [0.62–0.78].

## 4 Discussion

Our results showed that post‐NAC axillary ultrasound, although widely adopted, offers only moderate diagnostic performance closely related to the tumor subtype and to breast pCR. In the univariate analysis, the risk of false negatives is especially high in patients with ER+/Her2 tumors, lobular subtype and in those without breast cCR, raising caution against using AUS as the unique determinant for surgical de‐escalation. However, in the subsequent multivariate analysis, only the absence of breast cCR remained a significant predictor. Conversely, AUS performs better in Her2+ and ductal carcinomas, where its predictive value is more reliable [16]. In interpreting these findings, it's important to acknowledge that NAC regimens vary significantly by biomolecular subtype and directly affect pCR and nodal clearance. Although we adjusted for treatment regimen in our multivariable models, a degree of temporal confounding remains possible, particularly for patients with Her2+ disease. The progressive introduction and optimization of anti‐Her2 therapies in the early 2000s has markedly improved nodal response rates, and this evolution could partially account for the superior AUS performance observed in the subgroup. Recognizing these temporal and biological differences is essential when comparing post‐NAC imaging performance across heterogeneous patient populations.

Our findings also need to be considered against the background of the main trials that defined the limits of surgical de‐escalation after NAC: evaluating axillary de‐escalation after NAC demonstrates that, even optimized, SLNB carries a non‐negligible false‐negative rate [1, 2, 3]. Across these trials, dual tracer and retrieval of three or more sentinel nodes consistently improved accuracy, but residual nodal disease was not eliminated. It is therefore unlikely that AUS, used as a single modality, can reach a level of reliability higher than that of optimized sentinel surgery in this setting [1, 2, 3].

Large studies highlighted the challenges of accurately identifying residual nodal disease after NAC, even when sentinel lymph node biopsy is employed, with false‐negative rates (FNR) often exceeding 10% in selected subgroups. In ACOSOG Z1071, the overall FNR of SLNB after NAC was 12.6%, improving to nearly 6.8% only when the clipped node was retrieved [1, 24]. SENTINA similarly reported a FNR of 14.2% in patients converting from cN+ to ycN0, highlighting the challenges of accurate axillary restaging [3]. The SN FNAC trial showed that careful pathological assessment (including IHC) and technique can reduce the FNR to 8.4% (rising to nearly 13% not including IHC) [2]. More recently, early analyses from the AXSANA cohort show substantial inter‐institution variability in FNR, underscoring real‐world limitations [22]. These limitations led to the incorporation of strategies such as dual tracer mapping or TAD to improve accuracy [20, 21, 25]. Similarly, our findings underscore that AUS alone, while simple and accessible, cannot achieve the reliability required to guide omission of axillary dissection in high‐risk patients.

In addition, the biologically driven differences observed in our cohort—particularly the superior performance in Her2+ tumors—mirror the well‐documented chemosensitivity of this subtype and its higher rates of nodal clearance [11]. Conversely, the difficulty of interpreting AUS in lobular carcinomas and ER+/Her2‐ subtypes reflects the subtler morphologic changes and reduced chemotherapy responsiveness already described in earlier reports [12]. Moreover, technical issues such as marker loss during axillary surgery, recently highlighted in large prospective cohorts [23], further complicate the surgical management of these patients. Even in expert hands, marker loss compromises the retrieval of the clipped node during TAD, potentially reducing the reliability of this technique. This highlights that axillary management after NAC is not only a matter of imaging performance but also of surgical feasibility. Our results, together with AXSANA, reinforce that careful multidisciplinary evaluation—including imaging, surgical planning, and pathology—is needed to avoid both undertreatment and overtreatment.

AUS can serve as an initial tool, but multimodal strategies—incorporating MRI, PET/CT, or selective node localization techniques—are essential to safely reduce the burden of axillary surgery. Several groups have also investigated whether adding other imaging techniques to AUS can improve post‐NAC axillary assessment [26]. Turan and colleagues compared AUS, MRI, and 18F‐FDG PET/CT in patients with biopsy‐proven axillary metastases [27]. In their study, AUS was found to be the best single modality after NAC, whereas MRI and PET/CT alone were less accurate. When both AUS and PET/CT were positive, specificity and PPV resulted extremely high; when both were negative, a subgroup of patients emerged in whom SLNB rather than ALND could be reasonably considered [27].

Redaelli et al. [28] evaluated nodal response to chemo‐immunotherapy using RECIST 1.1 and found that changes in lymph node size on cross‐sectional imaging correlated only weakly with the final pathological nodal status. A relevant number of patients classified as radiological responders still had viable tumor in the axilla [28].

Malhaire et al. in a retrospective cohort of patients candidate for NAC highlighted that pre‐treatment axillary US and breast MRIs may improve prediction of post‐NAC nodal response [29].

Taken together, these data and our results indicate that imaging based on lymph node morphology, even when several modalities are combined, is useful to refine risk stratification but is not robust enough to replace surgical staging in initially node‐positive patients.

## 5 Conclusions

Post‐NAC AUS shows only moderate diagnostic accuracy for cN+ breast cancer patients, with its performance strongly influenced by tumor biology, histological subtype, and response to NAC. After NAC, AUS alone may be unreliable for restaging, with a high risk of false‐negative results, particularly in patients without a complete radiological or pathological response of the primary tumor. Although AUS is a widely available and non‐invasive imaging modality, our findings suggest that it should not be used alone to decide on omission of axillary surgery in initially node‐positive patients. A multimodal, biology‐informed approach, combining AUS with other imaging techniques and with optimized sentinel or targeted axillary procedures, is likely to be necessary to safely de‐escalate axillary treatment and to reduce the risk of both under‐ and overtreatment.

## Author Contributions

**Sara Albasini:** methodology, data curation, formal analysis, writing – original draft. **Camilla Rossetti:** data curation, writing – original draft. **Roberta Brancaccio:** methodology, data curation, formal analysis, writing – original draft. **Elena Orvieto:** data curation. **Daniela Bossi:** methodology, data curation, formal analysis, writing – original draft. **Matilde Pelizzola:** data curation. **Marta Truffi:** writing – original draft. **Serena Mazzucchelli:** writing – original draft. **Valentina Zanella:** writing – original draft. **Arianna Bonizzi:** writing – original draft. **Carlo Morasso:** writing – review and editing, methodology, writing – original draft. **Fabio Corsi:** conceptualization, supervision, writing – review and editing, methodology, writing – original draft.

## Funding

This research was partially funded by the Italian Ministry of Health (Ricerca corrente program).

## Ethics Statement

No. 2590/2021 Ethical Committee of Istituti Clinici Scientifici Maugeri, Pavia, Italy. Written informed consent was obtained for all patients.

## Conflicts of Interest

The authors declare no conflicts of interest.

## Data Availability

The data set generated and analysed during the present study is available from the corresponding author upon reasonable request.
